# Gastrointestinal transport of calcium and glucose in lactating ewes

**DOI:** 10.14814/phy2.12817

**Published:** 2016-06-07

**Authors:** Stefanie Klinger, Bernd Schröder, Anja Gemmer, Julia Reimers, Gerhard Breves, Jens Herrmann, Mirja R. Wilkens

**Affiliations:** ^1^ Department of PhysiologyUniversity of Veterinary Medicine Hannover, FoundationHannoverGermany

**Keywords:** Ca absorption, calcium, gastrointestinal tract, glucose, lactation, sheep

## Abstract

During lactation, mineral and nutrient requirements increase dramatically, particularly those for Ca and glucose. In contrast to monogastric species, in ruminants, it is rather unclear to which extend this physiological change due to increased demand for milk production is accompanied by functional adaptations of the gastrointestinal tract (GIT). Therefore, we investigated potential modulations of Ca and glucose transport mechanisms in the GIT of lactating and dried‐off sheep. Ussing‐chamber technique was applied to determine the ruminal and jejunal Ca flux rates. In the jejunum, electrophysiological properties in response to glucose were recorded. Jejunal brush‐border membrane vesicles (BBMV) served to characterize glucose uptake via sodium‐linked glucose transporter 1 (SGLT1), and RNA and protein expression levels of Ca and glucose transporting systems were determined. Ruminal Ca flux rate data showed a trend for higher absorption in lactating sheep. In the jejunum, small Ca absorption could only be observed in lactating ewes. From the results, it may be assumed that lactating ewes compensate for the Ca loss by increasing bone mobilization rather than by increasing supply through absorption from the GIT. Presence of SGLT1 in the jejunum of both groups was shown by RNA and protein identification, but glucose uptake into BBMV could only be detected in lactating sheep. This, however, could not be attributed to electrogenic glucose absorption in lactating sheep under Ussing‐chamber conditions, providing evidence that changes in jejunal glucose uptake may include additional factors, that is, posttranslational modifications such as phosphorylation.

## Introduction

During lactation, nutrient and mineral requirements increase dramatically. This physiological change due to the high demand of milk production cannot be compensated only by adjusting dietary intake. In order to enable increased absorption capacities, lactation is accompanied by morphological and functional adaptations of the maternal gastrointestinal tract (Hammond 1997). The daily energy and Ca requirements for maintenance of a sheep weighing 70 kg amount to 9.4 MJ and 2.4 g, respectively. The same animal in early lactation, assuming a milk yield of 2.5 kg, would need 22.9 MJ and 9.8 g Ca (National Research Council 2007). Failure to meet these requirements can result in typical peripartal diseases such as clinical and subclinical hypocalcemia and metabolic disorders related to negative energy balance (Brozos et al. 2011; Reinhardt et al. 2011; Mahrt et al. 2015).

For monogastric animals, it has been shown that lactation increases the efficiency of intestinal Ca absorption. In rats and mice, structures involved in active, transcellular Ca absorption, that is, the epithelial Ca channel TRPV6 (transient receptor potential vanilloid type 6), the cytosolic Ca binding protein CaBP‐D_9K_, and the basolateral Ca pump PMCA1b (plasma membrane Ca ATPase 1b), are upregulated in lactating animals (Zhu et al. 1998; Van Cromphaut et al. 2003; Douard et al. 2012). For ruminants, respective data on the expression of Ca transporting structures has not yet been obtained. Nevertheless, in balance studies, it has been demonstrated that the Ca net absorption in lactating ewes is significantly higher than in dried‐off sheep (Braithwaite et al. 1969). Similar results were obtained in experiments with dairy cows at the onset of lactation (Ramberg et al. 1970).

Although dry ruminants are fed high‐roughage diets, lactating animals have to be supplied with high‐concentrate mixtures to avoid negative energy balance. It has been shown in studies with different sources of carbohydrates incorporated in concentrate mixtures that ruminal starch degradation is no longer almost complete, but varies between 70 and 90% (Lebzien and Engling 1995; Noziere et al. 2014). The starch proportion escaping ruminal fermentation would subsequently be digested in the small intestine. Feeding growing goats with 60% barley grain caused luminal pH to decline in both rumen and colon and a greater colonic concentration of short‐chain fatty acids (SCFAs) compared with those fed 0 and 30% grain diets, and it was proposed that this may induce coordinated changes in the molecular response of the GIT's epithelium to increase systemic absorption of SCFAs (Metzler‐Zebeli et al. 2013). Therefore, current research aims at studying how to improve the efficiency of carbohydrate digestion in the GIT. It has been shown in vitro that the duodenal glucose uptake in dairy cows at an early stage of lactation may be higher compared to later stages of lactation (Okine et al. 1994). As for monogastric animals, it is known for ruminants that the transepithelial glucose transport consists of the Na^+^‐linked glucose transporter 1 (SGLT1) along a Na^+^‐gradient maintained by the activity of the basolateral located Na^+^/K^+^‐ATPase and the basolateral glucose extrusion by facilitated diffusion via the channel protein glucose transporter 2 (GLUT2).

SGLT1 is highly active in preruminant animals, but is downregulated in ruminating adults due to a lack of substrate in the upper small intestines because most glucose is fermented in the rumen (Scharrer 1976; Scharrer et al. 1979; Shirazi‐Beechey et al. 1991; Wood et al. 2000). However, it is uncertain to what extent and by which mechanisms the small intestine of ruminants is able to restore the glucose absorption capacity in response to increased luminal substrate availability in case of feeding concentrate mixtures with carbohydrates. Respective studies are inconsistent regarding the existence of such a restoration, but mainly regarding the underlying mechanisms. In general, the intestinal glucose absorption via SGLT1 can be regulated at different levels such as the mRNA expression, the amount of SGLT1 that is inserted into the apical membrane originating from intracellular storage vesicles as well as via altering the transporter activity by posttranslational modifications, for example, protein phosphorylation (Wright et al. 2011).

It has been demonstrated in sheep that changes in the number of SGLT1 in the apical membrane is not accompanied by an appropriate increase in mRNA expression, thus indicating a relevant role for SGLT1 translational or posttranslational modifications (Lescale‐Matys et al. 1993; Wright et al. 2011). In contrast, recent research indicates a certain correlation between RNA expression and protein function for cattle (Moran et al. 2014). Data on effects of feeding high‐starch diets to dairy cows are rare, but there is evidence that feeding a high‐starch diet does not lead to an increase in SGLT1 expression (Lohrenz et al. 2011). It could be shown in calves (Klinger et al. 2013a) and growing goats (Klinger et al. 2013b) that feeding carbohydrate‐rich diets leads to an increased glucose uptake into intestinal BBMV and it was hypothesized (Klinger et al. 2013a) that the regulation of SGLT1 activity via protein phosphorylation (Ishikawa et al. 1997; Vayro and Silverman 1999; Khoursandi et al. 2004) may play a role. This study focuses on the aspect that Ca and glucose transport capacities may be upregulated in lactating sheep due to hormonal adaptations and also higher substrate availability. In rats, it has been shown that luminal glucose increases Ca absorption via the voltage‐dependent Ca channel Ca_v_1.3 by an SGLT1‐dependent mechanism (Morgan et al. 2007). Thus, functional studies were carried out in Ussing‐chambers to determine ruminal and jejunal unidirectional Ca and mannitol flux rates, as a marker of the paracellular pathway, and electrophysiological properties in response to the mucosal addition of glucose. Isolated intestinal BBMV were used for kinetic characterization of Na^+^‐dependent glucose uptake. In addition, Ca and glucose transporting systems were also quantified on respective RNA and protein level.

## Material and methods

### Animals

The protocol for animal treatment was approved and supervised by the Animal Welfare Commissioner of the University of Veterinary Medicine Hannover according to the German Animal Welfare Law. The study was carried out with 10 multiparous sheep (East Friesian dairy sheep) aged between 2 and 5 years. Sheep were housed in a free‐stall barn at the Department of Physiology of the University of Veterinary Medicine Hannover, Foundation, Germany.

All animals had access to hay, minerals, and water ad libitum. Until parturition, the animals were group‐fed 7.5 g concentrate per kg body weight (BW) and day; Table 1; composition (% original substance) of hay (first number) and concentrate (second number) crude protein: 8.21/20.0; crude fat 1.52/2.60; crude fiber 26.5/11.0; crude ash 7.30/8.70; calcium 0.41/0.90; phosphorus 0.32/0.55; sodium 0.11/0.20, respectively. After parturition, the lambs were removed and milk yield was recorded for 20 consecutive days, by milking the animals three times daily at 0800, 1400, and 2000 h. The amount of concentrate to be fed to each sheep was calculated according to the milk yield of the previous milking (800 g kg^−1^ milk) to meet the requirements for dairy sheep recommended by the Society of Nutrition Physiology (GfE, Frankfurt, Germany). This resulted in an average daily concentrate intake of 32 g kg^−1^ BW. On day 21 postpartum, five lactating animals were killed. A further five sheep were dried‐off after 3 months of lactation. Concentrate was reduced to 25 g per 10 kg BW and 6 weeks after lactation had ceased, the animals were killed.

**Table 1:** Composition of buffer solutions used in Ussing‐chamber experiments. Concentrations given in mmol L^−1^ or in case of HCl and NaOH in mL L^−1^, respectively. Determination of pH at 38°C, buffers aerated with carbogen. As only ionized Ca is transported, its concentration was adjusted in the buffer solutions and determined using an electrolyte analyzer (Rapidlab 248, Chiron Diagnostrics GmbH, Fernwald, Germany). All chemicals were of analytical grade (Merck, Darmstadt, Germany or Sigma‐Aldrich Chemicals, St.Louis, Missouri)

Substance	Jejunum		Rumen	
	Serosal	Mucosal	Serosal	Mucosal
NaCl	113.6	113.6	55.5	57.0
KCl	5.4	5.4	5.0	5.0
CaCl_2_∙2H_2_O	1.2	1.2	2.2	1.45
Ca^2+^	0.9	0.9	0.8	0.8
MgCl_2_∙6H_2_O	1.2	1.2	1.2	1.2
NaHCO_3_	21.0	21.0	21.0	2.0
Na_2_HPO_4_∙2H_2_O	1.2	1.2	1.4	0.5
NaH_2_PO_4_∙H_2_O	–	–	1.4	2.3
Glucose	10.0	–	10.0	5.0
Na acetate∙3H_2_O	–	–	–	36.0
Na propionate	–	–	–	15.0
Na butyrate	–	–	–	9.0
Na gluconate	6.0	–	60.0	20.9
Mannitol	1.2	1.2	0.8	0.8
HEPES	7.0	20.0	–	–
1 N HCl	0.2	0.2	0.4	0.8
1 N NaOH	–	6.0	–	–
pH	7.42	7.42	7.52	6.50

### Sample collection

After captive bolt stunning and exsanguination from the carotid arteries, ruminal fluid and samples of the ventral ruminal sac and the jejunum were taken within 10 min postmortem. Prior to further handling, tissues were rinsed with ice‐cold saline. For Ussing‐chamber experiments, the jejunum was opened along the mesenteric line and kept in ice‐cold serosal buffer solution (Table 1) aerated with carbogen (95% O_2_/5% CO_2_) until the stripped mucosa was mounted in the chambers 20 min later. Ruminal mucosa was stripped immediately off the underlying muscle layers and kept in mucosal buffer solution (Table 1) aerated with carbogen and kept at 38°C until it was mounted in the Ussing‐chambers 15 min later. Tissues for the preparation of isolated BBMV were immediately frozen in liquid nitrogen and stored at −80°C for subsequent experiments, while tissues for qPCR and western blot analyses were frozen and stored accordingly after the removal of the serosa and adjacent muscle layers. Ruminal fluid was frozen after centrifugation (38,720 *g*, 20 min, 4°C) at −20°C.

### Measurements of Ca and short‐chain fatty acids in rumen fluid

Rumen fluid concentration of Ca was measured colorimetrically by a standard spectrometric technique (Sarkar and Chauhan 1967). Concentrations of acetate, propionate, and butyrate in rumen fluid were determined by gas chromatography. Following a first centrifugation at 48,000 *g* for 20 min, 1 mL of rumen fluid was acidified by adding 0.1 mL formic acid (98%) and centrifuged again at 40,000 *g* for 10 min in order to remove precipitated substrates. Gas chromatography (model 5890 II; Hewlett Packard, Boeblingen, Germany) was performed using Chromosorb WAW (mesh 80/100) with 20% neopentyl glycol succinate and 2% phosphoric acid as column matrix and helium as carrier gas followed by flame ionization detection (von Engelhardt and Sallmann 1972).

### Ussing‐chamber studies

For each segment of the gastrointestinal tract, epithelia were mounted between the two halves of six incubation chambers, each with an exposed area of 2.00 cm^2^ (rumen) or 1.13 cm^2^ (jejunum). Serosal and mucosal compartments of each Ussing‐chamber were connected to circulation reservoirs filled with respective buffer solutions (Table 1), maintained at 38°C by water jackets and continuously stirred and aerated with carbogen by a gas lift system.

A computer‐controlled voltage clamp device (Mussler Scientific Instruments, Aachen, Germany) was used to determine the transepithelial potential difference (PD_t_) with reference to the mucosal side, the tissue conductance (*G*_t_), and the short‐circuit current (*I*_sc_). All experiments were carried out under short‐circuit current conditions. This means that a direct current was applied to the mucosa setting PD_t_ to 0 mV and accounted for the offset potential and the resistance of the bathing solution. The current was adjusted to changes in electrical activity of the mucosa every 6 sec. *G*_t_ values were calculated from bipolar pulses of 0.2 sec duration and 100 *μ*A amplitude, which were superimposed on the short‐circuiting current. The displacement in potential after 0.1 sec duration induced by these pulses and the pulse amplitude were used to calculate *G*_t_ and PD_t_ on the basis of Ohm's law as described earlier (Schröder et al. 1991).

Viability was verified by the secretory response of the jejunum to forskolin (10 *μ*mol L^−1^, serosal side) and the collapse of the ruminal PD_t_ induced by ouabain (100 *μ*mol L^−1^, serosal side) at the end of the experiments.

Unidirectional Ca and mannitol flux rates were simultaneously determined using ^45^Ca and ^3^H‐mannitol as tracers. Mannitol was used as a marker for fluxes via the paracellular pathway (Matysiak‐Budnik et al. 2004). For each radioisotope, 185 kBq (5 *μ*Ci) were added to the chambers as ^45^CaCl_2_ (specific activity > 370 GBq/g; PerkinElmer Life Sciences, Rodgau‐Jürgensheim, Germany) and ^3^H‐mannitol, respectively, either to the serosal or to the mucosal side. Following an equilibration period (30 min), eight samples (P1–P8) of 500 *μ*L each were taken at intervals of 15 min (jejunum) or 20 min (rumen). The volume was immediately replaced by the respective buffer solution. Radioactivity of the samples was measured using a liquid scintillation counter (Tricarb Packard, Dreieich, Germany) and flux rates from the mucosal to the serosal side (*J*_ms_) and from the serosal to the mucosal side (*J*_sm_) were calculated from the rate of tracer appearance using standard equations (Schultz and Zalusky 1964). To determine net flux rates (*J*_net_), *J*_sm_ was subtracted from the respective *J*_ms_. For the jejunum, mean values of the three flux rates calculated from the first four samples were used to define basal Ca and mannitol transport. Subsequently, glucose was added to the mucosal side to a concentration of 10 mmol L^−1^. The effects of mucosal glucose were calculated from the difference of flux rates determined from P4–P8 and the basal flux rates.

### BBMV isolation and preparation of crude membranes and cytosol

Aside from the buffer for the final homogenization step and resuspension (10 mmol L^−1^ HEPES, 100 mmol L^−1^ mannitol, 100 mmol L^−1^ KCl, pH 7.4), BBMV were prepared using a Mg^2+^‐EGTA precipitation method as described previously (Schröder and Breves 1996). Crude membranes and cytosol were prepared as described previously (Schröder et al. 2001; Wilkens et al. 2011). Protein concentrations were determined using Bio‐Rad Protein Assay (Bio‐Rad Laboratories GmbH, Munich, Germany) with bovine *γ*‐globulin as standard.

### Enrichments of apical membranes (AP and Na^+^/K^+^‐ATPase activities)

Enrichment and purity of BBMV were determined as the ratio of enrichment of the apical marker alkaline phosphatase activity (Ohmori 1937) and the enrichment of basolateral Na^+^/K^+^‐ATPase activity (Mircheff and Wright 1976) in the final vesicle suspension (P2) compared to the primary homogenate (P1; P2: P1 = relative enrichment factor).

### ^3^H‐glucose/glucose uptake studies

^3^H‐glucose/glucose uptake experiments were carried out as previously described (Klinger et al. 2013a). Na^+^‐dependent ^3^H‐glucose was determined as the difference of ^3^H‐glucose uptake in the presence of Na^+^ (Na^+^‐containing buffer) and the absence of Na^+^ (K^+^‐containing buffer). Uptake was measured at room temperature for 15 sec at extravesicular glucose concentrations in eight steps from 0.01 to 1.5 mmol L^−1^. The Na^+^‐dependent component of ^3^H‐glucose uptake was analyzed according to Michaelis and Menten to calculate the maximal uptake velocity (*V*_max_) as an estimate of SGLT1 number and the substrate concentration at which the uptake rate is at half‐maximum (Michaelis–Menten constant, *K*_m_) as a measure of the glucose affinity of SGLT1.

### Western blot analysis

After sample denaturation (see below), samples were separated by SDS‐PAGE and transferred to nitrocellulose membranes using the tank blot procedure.

For the detection of SGLT1 expression in BBMV 8 *μ*g protein were heat‐denatured (15 min, 40°C, denaturing buffer containing 10% DTT). Blocked membranes (5% milk powder/TBST for SGLT1, 90 min, RT) were incubated with rabbit anti‐SGLT1 antibody (abcam^®^ , Cambridge, UK; ab 14686, 1:1,000 in 5% milk/TBST, 12 h, 4°C) and subsequently incubated with HRP (horseradish peroxidase) conjugated anti‐rabbit IgG (Sigma A9169, 1:30,000 in 5% milk/TBST, 1 h, RT). GLUT2 expression (apical membranes/crude membranes, 20 *μ*g protein, denatured for 20 min, 70°C, denaturing buffer/10% DTT) was detected accordingly with the primary antibody (rabbit anti‐GLUT2, antibodies‐online.com ABIN 739124) being used in a 1:500 dilution.

To determine the expression of Na^+^/K^+^‐ATPase, crude membranes were heat‐denatured (10 *μ*g protein, 20 min, 70°C, denaturing buffer/10% DTT). Membranes were incubated with mouse anti‐Na^+^/K^+^‐ATPase antibody (Enzo Life Sciences ALX‐804‐082, 1:10,000 in 5% milk/PBST, 12 h, 4°C) and with the corresponding secondary antibody (HRP conjugated anti mouse IgG, Sigma A2304, 1:20,000 in 5% milk/PBST, 1 h, RT). After incubation with the respective primary and HRP‐conjugated secondary antibodies, HRP was visualized by chemiluminescence (Pierce, Thermo Scientific, Waltham, MA) using a ChemiDoc system (Bio‐Rad). Protein expression was calculated with the Quantity one software (Bio‐Rad). Equal loading was confirmed by analyzing the ratio of the specific signal and the signal after Ponceau 2R staining of the respective lane as analyzed with Image J. Western blot analyses of crude membranes for TRPV6 and PMCA1/4, and determination of CaBP‐D_9K_ in the cytosolic fraction were done as described earlier (Schröder et al. 2001; Wilkens et al. 2011).

### RNA isolation and quantitative RT‐PCR

Total RNA was isolated using the RNeasy Mini‐Kit (Qiagen, Hilden, Germany) according to the manufacturer's protocol. Concentration and quality of the RNA were determined by UV absorbance and 200 ng was reverse transcribed using TaqMan‐Reverse Transcription Reagents (Applied Biosystems, Darmstadt, Germany). For quantification of the housekeeping gene *β*‐actin, TRPV6, PMCA1b, and Ca_v_1.3 gene‐specific TaqMan^®^ primers and probes (Table 2) were purchased from TIB MOLBIOL (Berlin, Germany). Reaction mixtures (20 *μ*L) contained TaqMan Universal PCR Master Mix (Life Technologies, Darmstadt, Germany), specific primers (300 nmol L^−1^, specific probe (100 nmol L^−1^), and 16 ng reverse transcribed RNA. PCR products were amplified (50°C, 2 min; 95°C, 10 min; 40 cycles of 95°C, 15 sec and 60°C, 1 min) and analyzed on a real‐time PCR cycler (CFX96^TM^, Bio‐Rad).

**Table 2:** Primers and probes used for TaqMan^®^ and SYBR green^®^ assays

Gene	Sense and antisense primers, probes (5′→ 3′)	Source
*β*‐actin	CTCAGAGCAAGAGAGGCATCC GCAGCTCGTTGTAGAAGGTGTG FAM‐CAAGTACCCCATTGAGCACGGC ‐TMR	This study
TRPV6	TGATGGGAGACACTCACTGG GCAGCTTCTTCTCCAGCATC FAM‐TGGCTACAACCTGCGCCCT ‐TMR	Wilkens et al. (2009)
PMCA1b	GGTATTGCTGGAACTGATGTAGCTAA CGTCCCCACATAACTGCTTT FAM‐CAATGCTTGTAAAATTGTCATCCGTGAGA‐TMR	Wilkens et al. (2011)
Cav1.3	ATGATGAACATCTTCGTGGGCT GAAAGGCGAGGAGTTCACC FAM‐CTGGACAAAAATCAGCGTCAGTGTGTT‐BBQ	This study
CaBP‐D_9K_	GCCAAAGAAGGTGATCCAAA CCAACACCTGGAATTCTTCG	Elfers et al. (2015)
SGLT1	CATCCTGACTGGGTTTGCTT ATGGTCAGCCCAAAGATGAG	This study
GLUT2	TGTTGTCACGGGCATTCTTA CAAGCTTTTCTTTGCCTTGG	This study
Na^+^/K^+^‐ATPase	TGGAACTCGGAGAAGAAGGA GCCACTCGGTCCTGATATGT	This study

Expression of CaBP‐D_9K_, SGLT1, GLUT2, and Na^+^/K^+^‐ATPase was determined using SYBR Green^®^ PCR assays. Specific primers (Table 2) were purchased from Life Technologies. Reaction mixtures (20 *μ*L) contained KAPA SYBR FAST Universal Master Mix (PEQLAB Biotechnologie GmbH, Erlangen, Germany), specific primers (200 nmol L^−1^), and 16 ng reverse transcribed RNA. PCR products were amplified (95°C, 3 min; 40 cycles of 95°C, 10 sec and 60°C, 30 sec) and detected on a real‐time PCR cycler (CFX96TM, Bio‐Rad). The thermal profile for melt curve determination began with an incubation of 10 min at 55°C with a gradual increase in temperature (0.5°C/10 sec) to 95°C.

Absolute copy numbers were determined using calibration curves generated with cloned PCR fragment standards as described elsewhere (Wilkens et al. 2009). Efficiency of the PCR assays tested in advance for dilutions of renal cDNA of both species and cloned standard ranged from 90 to 105%. Specificity of the amplicons was verified using NCBI Blast (http://blast.ncbi.nlm.nih.gov/Blast.cgi). Parallel PCR assays for each gene target were performed with cDNA samples, genomic standards, and a no‐template control. Each series of experiments was carried out twice.

### Statistical analyses

Comparison of electrophysiological parameters was carried out by analysis of variance for repeated measurements followed by Fisher least significant difference test for comparison of the estimated marginal means. These statistical analyses were performed using SPSS 21.0 (SPSS, Chicago, Illinois). Data on short‐chain fatty acid concentration, RNA, and protein expression were analyzed by GraphPad Prism 6 (GraphPad Software, San Diego, California). Means were tested for their deviation from zero. If means from both experimental groups were significantly different from zero, they were compared by unpaired *t*‐test. Otherwise, a deviation of the remaining mean from zero was accepted to reflect a significant difference between the groups. All values are presented as means ± SEM. In figures, the following symbols were used: (*) *P* < 0.1, *P < 0.05, **P < 0.01, ***P < 0.001.

## Results

### Concentrations of Ca and short‐chain fatty acids in rumen fluid

Ca concentrations in rumen fluid of lactating ewes were significantly greater than in dried‐off animals (3.85 ± 0.68 mmol L^−1^ vs. 1.98 ± 0.33 mmol L^−1^, *P* < 0.05, *N* = 5). Concentrate feeding did not affect the concentration of total SCFAs (dried‐off 74.2 ± 6.1 vs. lactating 79.6 ± 2.5 mmol L^−1^, *N* = 5) including acetate (dried‐off 54.0 ± 4.9 vs. lactating 50.6 ± 1.7 mmol L^−1^). On the other hand, propionate (dried‐off 13.0 ± 1.0 vs. lactating 18.3 ± 0.9 mmol L^−1^, *P* < 0.01) as well as butyrate (dried‐off 5.8 ± 0.5 vs. lactating 9.1 ± 1.5 mmol L^−1^, *P* < 0.1) concentrations increased, thus resulting in a significant increase in the propionate/acetate ratio (C3/C2 ratio: dried‐off 0.24 ± 0.01 vs. lactating 0.37 ± 0.03, *P* < 0.001). This indicates that concentrate feeding markedly increased propionate availability and this is in accordance with similar findings in goats fed increasing amounts of barley grain (Metzler‐Zebeli et al. 2013).

### Ussing‐chamber studies

**Figure 1:**
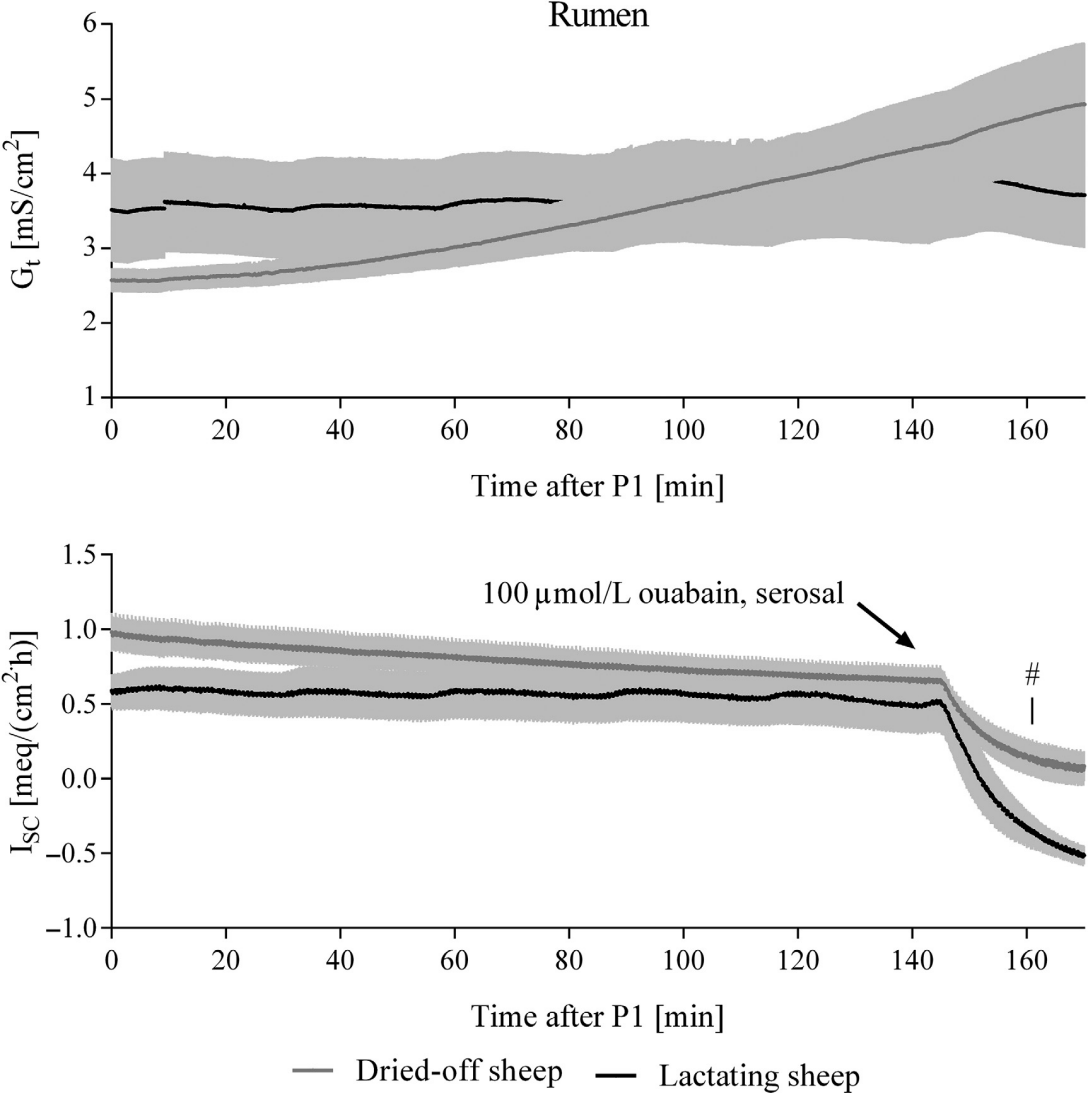
Time courses of tissue conductances (*G*_t_) and short‐circuit currents (*I*_sc_) recorded during incubation of ruminal preparations in Ussing‐chamber experiments. Analysis of variance (ANOVA) for repeated measurements revealed effects of time (*P *< 0.001) and a trend for an interaction of time and lactation (*P *< 0.1) for *G*_t_. Short‐circuit currents were influenced by time (*P *< 0.05) and by an interaction of time and lactation (*P *< 0.05), too. In addition, the decrease in *I*_sc_ within 20 min after addition of ouabain differed significantly (0.53 ± 0.08 meq/[cm^2 ^h] vs. 0.84 ± 0.08 meq/[cm^2 ^h], *P *< 0.05, marked by #). Means ± SEM,* N *= 5.

Rumen: While *G*_t_ and *I*_sc_ for preparations from lactating sheep remained stable throughout the incubation period, an increase in *G*_t_ accompanied by a small decrease in *I*_sc_ could be found for tissues from dried‐off animals (Fig. 1). The change in *I*_sc_ induced by ouabain was more pronounced in tissues from lactating sheep. A trend for greater *J*_ms_ of Ca and mannitol as well as for *J*_net_ of Ca indicating active Ca transport was revealed in lactating sheep, and *J*_sm_ flux rates are representing passive fluxes (Table 3).

**Table 3:** Ruminal and jejunal Ca and mannitol flux rates and respective changes after addition of 10 mmol L^−1^ glucose to the mucosal side of the epithelium (Δ glucose) determined in the absence of any electrochemical gradient in dried‐off and lactating sheep (nmol/[cm^2 ^h])

		Dried‐off sheep	Lactating sheep	Student′s *t* test
Rumen:	*J*_ms_ Ca	7.52 ± 0.54	9.60 ± 0.83	*P* < 0.07
	*J*_sm_ Ca	2.07 ± 0.44	2.05 ± 0.34	n.s.
	*J*_net_ Ca	5.45 ± 0.86	7.56 ± 0.66	*P* < 0.09
	*J*_ms_ Man	2.33 ± 0.58	4.41 ± 0.81	*P* < 0.07
	*J*_sm_ Man	3.71 ± 0.71	5.31 ± 0.67	n.s.
	*J*_net_ Man	−1.39 ± 0.36	−0.90 ± 0.23	n.s.
Jejunum: basal flux rates	*J*_ms_ Ca	40.3 ± 5.88	17.4 ± 3.87	*P* < 0.05
	*J*_sm_ Ca	41.6 ± 5.87	15.6 ± 3.88	*P* < 0.01
	*J*_net_ Ca	(−1.22 ± 4.13)	1.74 ± 0.58	n.a.*
	*J*_ms_ Man	40.9 ± 6.41	41.5 ± 8.53	n.s.
	*J*_sm_ Man	41.8 ± 6.01	31.3 ± 6.87	n.s.
	*J*_net_ Man	(−0.95 ± 2.25)	10.1 ± 2.08	n.a.*
Jejunum: Δ glucose	*J*_ms_ Ca	10.2 ± 3.13	8.79 ± 2.62	n.s.
	*J*_sm_ Ca	11.9 ± 3.68	7.49 ± 2.74	n.s.
	*J*_net_ Ca	(−1.74 ± 0.84)	(1.31 ± 1.00)	n.a.
	*J*_ms_ Man	11.9 ± 2.27	14.6 ± 3.24	n.s.
	*J*_sm_ Man	7.17 ± 1.95	6.77 ± 2.03	n.s.
	*J*_net_ Man	4.76 ± 1.99	7.79 ± 3.37	n.s.

*J*_ms_: unidirectional flux rate from mucosal to serosal, *J*_sm_: unidirectional flux rate from serosal to mucosal, *J*_net_ _=_ *J*_ms_ – *J*_sm_; *N *= 5; *n *= 3 (for each direction); means ± SEM; n.s. not significant; n.a. Student′s *t*‐test was not applied because at one of the mean fluxes (n.a.*) or both were not significantly different from the hypothetical value 0 (values given in brackets), Man mannitol, n.s. not significant.

**Figure 2:**
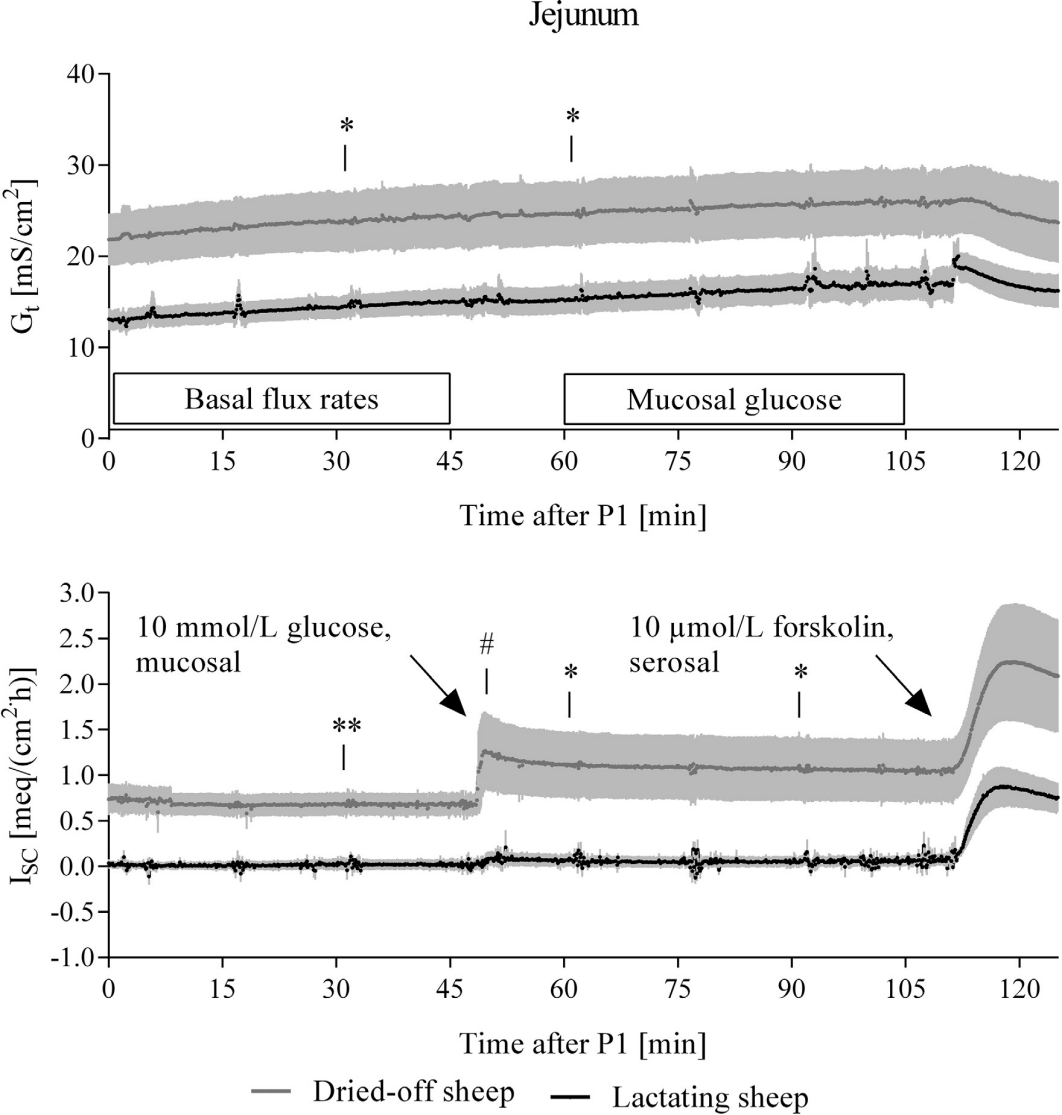
Time courses of tissue conductances and short‐circuit currents (*I*_sc_) recorded during incubation of jejunal preparations in Ussing‐chamber experiments. Analysis of variance (ANOVA) for repeated measurements revealed effects of time (*P *< 0.05) and lactation (*P *< 0.1) for *G*_t_. Short‐circuit currents were only significantly influenced by lactation (*P *< 0.05). Asterisks indicate significant differences revealed by comparison of estimated marginal. Addition of glucose to the mucosal side of the epithelia induced a significant increase in *I*_sc_ (0.57 ± 0.34 meq/[cm^2 ^h], marked by #) only in dried‐off sheep. Samples for determination of basal flux rates (P1–P4) were taken immediately after equilibration of the epithelia. Sampling for determination of glucose effects (P5–P8) was done after the increase of mucosal glucose concentration followed by another equilibration period of 15 min. Means ± SEM,* N *= 5.

Jejunum: Both, *G*_t_ and *I*_sc_ detected in preparations from lactating sheep were significantly lower in comparison to these electrophysiological parameters of dried‐off animals. Addition of glucose to the mucosal compartment induced a significant increase in *I*_sc_ in preparations from dried‐off, but not in those from lactating ewes (Fig. 2). While no significant differences could be found for unidirectional mannitol flux rates, unidirectional Ca flux rates were significantly greater in dried‐off sheep. But, as *J*_ms_ exceeded *J*_sm_ only in lactating ewes, a positive *J*_net_ for both, Ca and mannitol, could therefore only be detected in the lactating animals. In both groups, addition of glucose increased *J*_ms_ and *J*_sm_ of Ca to the same extent resulting in no detectable difference in *J*_net_. With respect to mannitol, glucose induced *J*_ms_ of mannitol stronger than *J*_sm_ leading to a positive *J*_net_ irrespective of the group (Table 3).

### AP and Na^+^/K^+^‐ATPase activities and BBM enrichments

The relative enrichments of the apical marker AP and the basolateral marker Na^+^/K^+^‐ATPase were not different between the two groups. Accordingly, the BBMV enrichment expressed as the ratio of these two values was not affected (data not shown). However, differences were observed with respect to absolute values (Table 4). Both the activities of AP and Na^+^/K^+^‐ATPase were higher in the P1 fraction from lactating animals. Since the protein content of the P1 fraction was significantly higher for lactating animals than for dried‐off animals, the differences in the volume activities are even more pronounced.

**Table 4:** Summary of functional data from BBMV uptake experiments (*V*_max_, nmol/[mg∙20 sec]) and *K*_m_ (mmol L^−1^) and BBMV characteristics (protein content, g L^−1^, enzyme volume activity (U L^−1^) and enzyme‐specific activity (U g^−1^), n.d. not detectable

	Dried‐off sheep	Lactating sheep	Student′s *t* test
*V*_max_	–	1.29 ± 0.16	–
*K*_m_	–	0.06 ± 0.01	–
Protein content	2.77 ± 0.18	3.82 ± 0.13	0.0015
Volume activity AP	5605 ± 1080	18282 ± 4414	0.0236
Volume activity Na^+^/K^+^‐ATPase	156.6 ± 18.40	375.4 ± 32.55	0.0004
Specific activity AP	2096 ± 425.1	4801 ± 1197	0.0659
Specific activity Na^+^/K^+^‐ATPase	56.70 ± 5.798	103.0 ± 10.23	0.0043

### Glucose uptake into isolated jejunal BBMV

**Figure 3:**
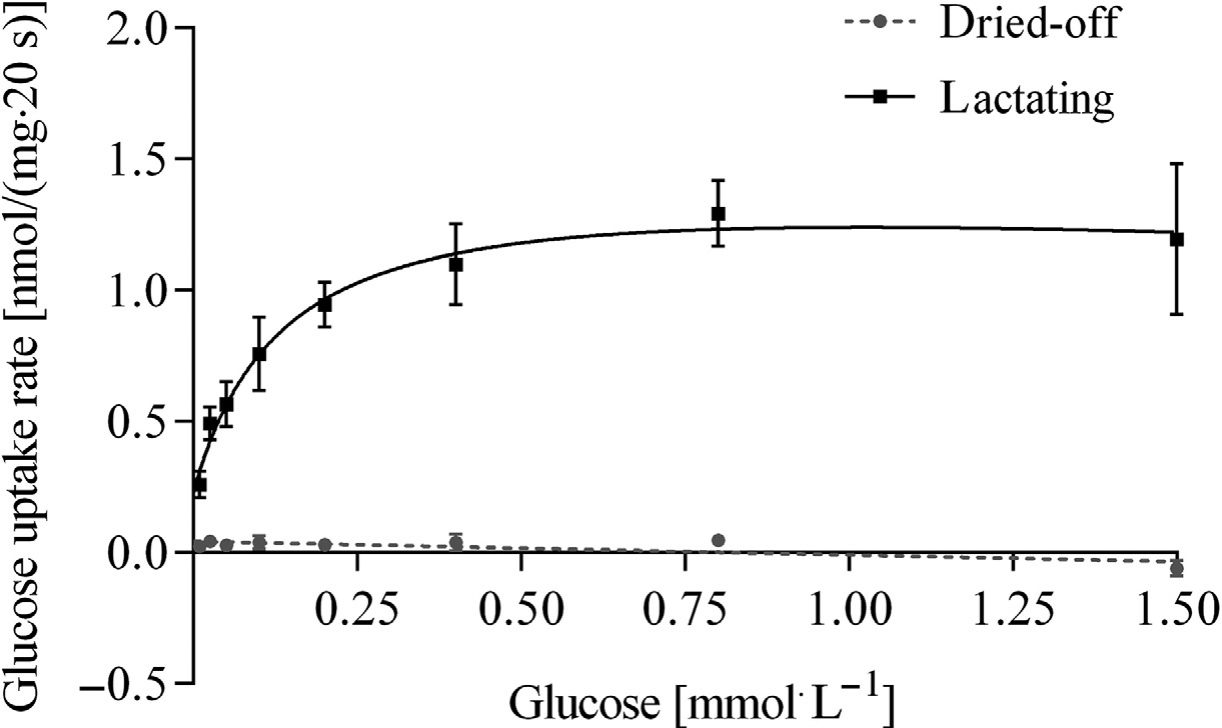
Na^+^‐dependent glucose uptake rates as a function of extravesicular glucose concentration into isolated jejunal BBMV from dried‐off and lactating sheep. Means ± SEM,* N* = 5.

Samples from four animals per group were used for the determination of apical glucose uptake and the calculation of kinetic parameters. Brush‐border membrane vesicles prepared from the jejunum of lactating sheep, showed a significant ^3^H‐glucose uptake characterized by a *V*_max_ of 1.29 ± 0.16 nmol/(mg∙20 sec) and a *K*_m_ of 0.06 ± 0.01 mmol L^−1^ (Table 4). Only a negligible ^3^H‐glucose uptake into BBMV prepared from dried‐off animals was detectable, if at all (Fig. 3). Since in part even negative values were measured it has to be stated, that the ^3^H‐glucose uptake into BBMV from this group was below the detection limit and has therefore assumed to be near zero for which reason no statistical comparison of the means was carried out.

### RNA and protein expression

**Figure 4:**
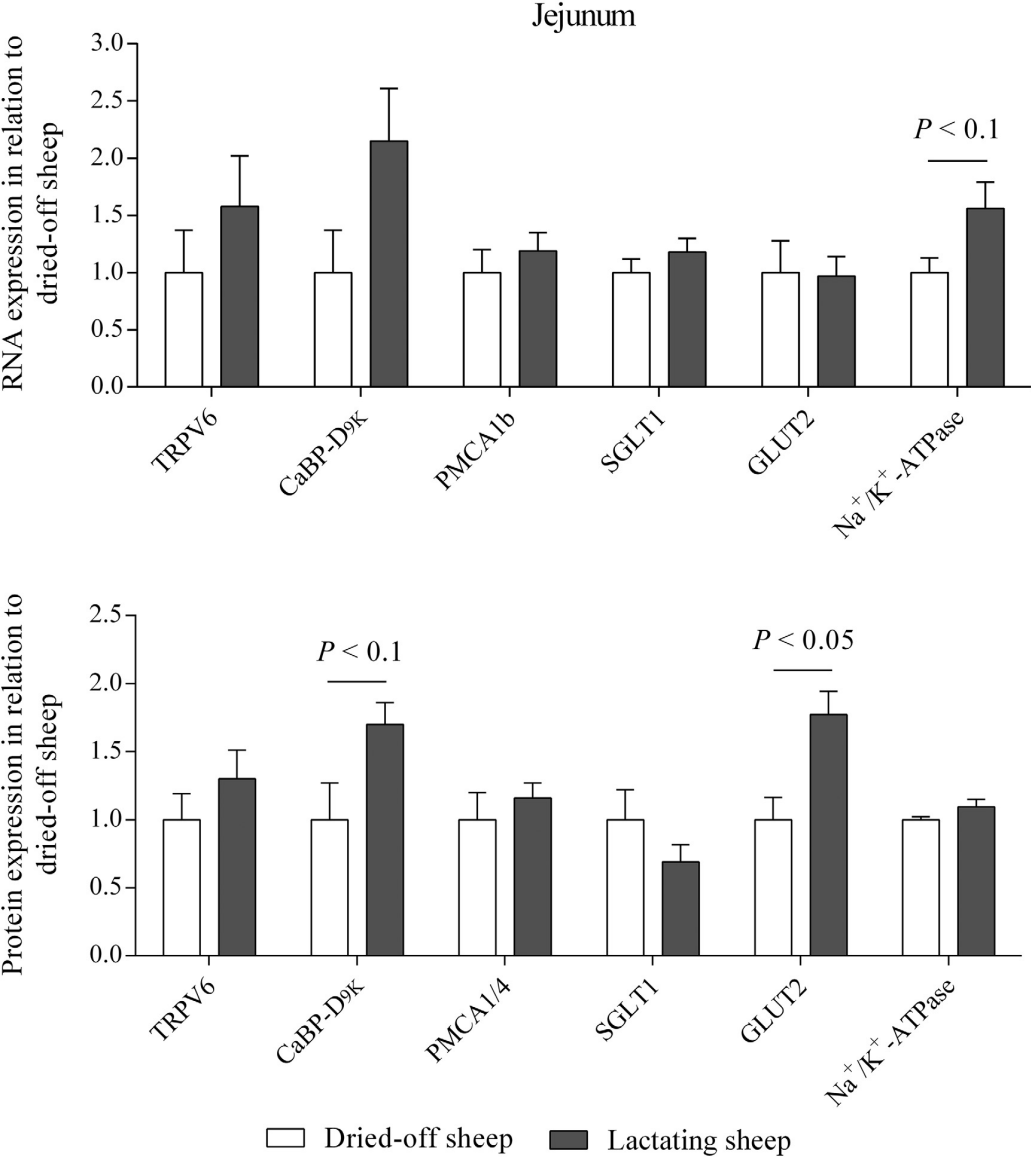
RNA (upper panel) and protein expression (lower panel) of TRPV6, CaBP‐D_9k_, PMCA1b, and PMCA1/4, respectively, as well as SGLT1, GLUT2, and Na^+^/K^+^‐ATPase in jejunal tissues of sheep either lactating (21st day p.p.) or after having been dried‐off for six weeks. *N *= 5, means ± SEM, results of Student′s *t*‐test are given within the figure.

**Figure 5:**
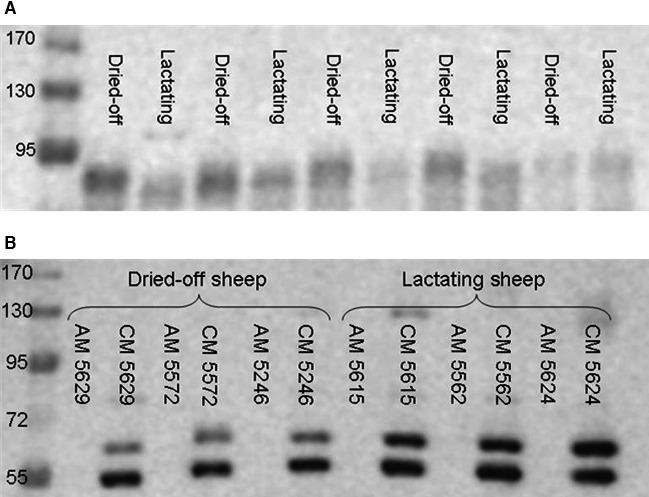
Western blot of SGLT1 (A) in apical membrane preparations and GLUT 2 (B) in apical membrane preparations (AM) and crude membrane preparations (CM).

Expression of Ca_v_1.3 was too weak to be quantified. Analyses of ruminal tissue for expression of PMCA1b and Na^+^/K^+^‐ATPase revealed no differences between dried‐off and lactating ewes (data not shown). Data on jejunal RNA and protein expression are summarized in Figure 4. Although expression of Ca transporting structures was greater in lactating animals, these results could not be verified statistically aside from a trend for higher CaBP‐D_9K_ protein expression. Although RNA expression for SGLT1 and GLUT2 did not differ between the groups, a trend for greater expression of Na^+^/K^+^‐ATPase in lactating sheep could be revealed, but no differences were detected for the Na^+^/K^+^‐ATPase protein expression. The amount of SGLT1 protein in apical membranes did not differ between the groups as well, but the detected bands seem to differ in their running behavior with a tendency toward higher bands for dried‐off sheep as shown in Figure 5. For GLUT2, two bands were detected. No antigenic peptide is available for the antibody used but the upper band is supposed to represent GLUT2 due to its height of approximately 60 kDa. Signals for GLUT2 protein were detectable in crude membrane preparations but not in apical membrane preparations (Fig. 5). The upper band was more abundant in crude membranes from lactating animals.

## Discussion

### Gastrointestinal Ca transport

In rodents fed a diet adequate in Ca, intestinal Ca absorption capacity as well as expression of respective Ca transporting proteins is stimulated during pregnancy and lactation (Zhu et al. 1998). As TRPV6 is upregulated in lactating VDR knockout mice, too (Van Cromphaut et al. 2003), this adaptation seems to be partly independent of an action of vitamin D. Interestingly, this study revealed a trend for greater ruminal Ca absorption in lactating sheep. Previous results from sheep and goats with dietary Ca restriction or treated with supraphysiological doses of calcitriol suggest that the so far unidentified ruminal Ca transport mechanism is also independent of vitamin D (Wilkens et al. 2011, 2012).

In lactating sheep, neither the expression of PMCA1b nor of Na^+^/K^+^‐ATPase was affected. However, differences could be found in respect to the collapse of the ruminal PD_t_ after addition of ouabain. Thus, it can be speculated that altered ion fluxes are involved in the adaptation of the rumen to lactation and/or the high‐luminal substrate and mineral concentrations when lactating sheep are kept on a ration calculated to meet the animals′ enhanced demands. Several studies have shown before that a high intake of concentrate alters electrophysiological properties as well as Na and Ca flux rates across the ruminal epithelium (Uppal et al. 2003a,b; Lodemann and Martens 2006). Therefore, it cannot be concluded from this study whether the observed effects are caused by lactation itself or by factors mediated by the respective feeding regime.

In the jejunum, a significant *J*_net_ of Ca could only be found in epithelia from lactating ewes. However, the flux was very small and the physiological relevance remains questionable. This finding is in line with the minor changes in CaBP‐D_9K_ protein expression and results from former studies demonstrating that the ovine intestine does not adapt to challenges of Ca homeostasis to the same extent that has been shown for monogastric species and also for goats (Wilkens et al. 2011, 2012). From the changes of plasma bone markers around parturition, it can be assumed that in comparison to goats, lactating ewes compensate for the Ca loss in milk by increasing bone mobilization for longer than by substantial stimulation of intestinal absorption (Wilkens et al. 2014).

In contrast to dried‐off animals, lactating ewes showed a significant *J*_net_ of mannitol indicating greater active absorption of nutrients and ions generating an osmotic gradient. Therefore, the observed *J*_net_ of Ca in lactating sheep might also be partly caused by solvent drag‐driven paracellular transport (Karbach 1992). A prolactin‐induced paracellular Ca transport has already been demonstrated for the duodenum of the rat (Tanrattana et al. 2004).

It was also shown that prolactin can induce transcellular Ca transport via Ca_v_1.3 (Thongon et al. 2009), and in addition, Ca_v_1.3 and glucose uptake via SGLT1 seem to be linked in the rat (Kellett 2011). However, against the background of its very low level of expression (data not shown) and the lack of a glucose‐induced increase in *J*_net_ of Ca, a physiologically relevant role of Ca_v_1.3 in intestinal Ca absorption in sheep is unlikely.

### Intestinal glucose transport

It was hypothesized that nutrient transport capacities, particularly those for glucose absorption, would be greater in lactating sheep than in dried‐off animals due to hormonal adaptations (e.g., prolactin) and higher substrate availability in the intestinal lumen. An increase in intestinal glucose concentration could be expected since concentrate intake was about fourfold higher during the lactation period. Under this assumption, a higher capacity for intestinal glucose transport can be anticipated. This may be supported by the findings of Metzler‐Zebeli et al. (2013) demonstrating that high‐grain diets can significantly increase concentrations of SCFAs in the colon of goats.

In Ussing‐chamber experiments, addition of glucose to the mucosal compartment normally induces a sudden increase in short‐circuit current which is related to electrogenic glucose absorption via SGLT1. Surprisingly, in this study, addition of glucose led to an increase in *I*_sc_ only in dried‐off animals but not in lactating sheep. According to the concept published by Kellett and co‐workers, high‐luminal concentrations of glucose can result in electroneutral glucose absorption by insertion GLUT2 into the apical membrane (Kellett et al. 2008). However, in this study GLUT2 could not be detected in apical membranes by western blot analysis.

In contrast to the results obtained from Ussing‐chamber studies, glucose uptake studies with isolated jejunal BBMV of lactating animals clearly showed a Na^+^‐dependent glucose uptake indicating activity of SGLT1 which was completely absent in dried‐off ewes. Interestingly, neither RNA nor protein expression of SGLT1 differed between the two groups. In crude membrane preparations, a greater protein abundance of GLUT2 was found in lactating sheep. This is consistent with the effects of a high‐carbohydrate diet in rats and may indicate a higher basolateral glucose exit capacity (Cheeseman and Harley 1991). The hypothesis of a greater nutrient and ion absorption in lactating ewes is further corroborated by the greater activity and RNA expression of the Na^+^/K^+^‐ATPase and the greater *J*_net_ of mannitol indicating solvent drag induced water absorption. Even the lower basal *I*_sc_ in lactating animals may be interpreted toward a higher overall transport activity of the epithelium since it has been shown in studies on total parenteral nutrition that the distribution of nutrients between the apical and the basolateral membrane is crucial for the pattern of electrogenic transport processes (Miura et al. 1992; Inoue et al. 1993; Peterson et al. 1996; Yang et al. 2003). Therefore, the basal differences in the *I*_sc_ as a measure of transepithelial net ion transport may also point to a higher transport activity in the small intestines of lactating animals.

Whether this altered transport properties in lactating sheep are due to the different composition of the digesta or due to hormonal changes regulating the intestinal properties (as elevated prolactin levels) cannot be differentiated based on the data of this study.

From these results, two main questions arise: (1) Why is the obvious presence of active SGLT1 in the apical membrane of enterocytes from lactating animals demonstrated by western blot analysis and uptake experiments not accompanied by an increase in *I*_sc_ after mucosal glucose addition in the Ussing? (2) Why is SGLT1 in the apical membrane of dried‐off animals` enterocytes as detected by western blot not able to mediate a significant Na^+^‐dependent glucose uptake into BBMV although electrogenic glucose absorption can be demonstrated in the Ussing‐chamber?

With regard to question 1, it has to be taken into account that not only the abundance of nutrient transporters but also the maintenance of both, the driving force and the membrane potential, is crucial for the absorption of a particular nutrient. Therefore, the composition of enterocytes in a highly absorptive state should also vary regarding for example the expression and activity of ion channels and ion pumps involved. Beside the Na^+^‐gradient maintained by the activity of Na^+^/K^+^‐ATPase, the membrane potential deserves attention since this is a major factor not only for Na^+^‐coupled glucose absorption but also for absorptive and secretory processes in general.

Both the respective driving forces and the individual activity of several transport proteins are dependent on either the transepithelial or the transmembrane potential difference across the apical or the basolateral membrane. The absorption of cations (Na^+^‐ or H^+^‐coupled symport transport systems) and the secretion of anions (Cl^−^ secretion) cause a depolarization of the apical membrane. Since the voltage‐dependent action of transport systems such as SGLT1 (Umbach et al. 1990), PepT1 (Mackenzie et al. 1996) or Na^+^‐dependent phosphate transporters (Forster et al. 2013) has to be ensured, it is essential to counteract this depolarization by compensating, hyperpolarizing ion currents.

It is known that in leaky epithelia as the jejunum, the depolarization is compensated for by an increase in the basolateral K^+^‐conductance in order to repolarize the membrane potential, whereas in tighter epithelia as the colonic mucosa, apical K^+^‐channels are necessary since basolateral ion currents are not sufficient to affect the transmembrane potential. It has been discussed that this may also apply to leaky epithelia under certain conditions (Warth and Barhanin 2003; Heitzmann and Warth 2008).

No evidence for apical K^+^‐secretion was found in the rat proximal jejunum (Cermak et al. 1999). However, apical K^+^‐secretion via KCNQ1 is involved in renal glucose resorption (Vallon et al. 2001), and in addition, it has been shown in mice that the knockout of KCNQ1 impairs the intestinal absorption of glucose and phenylalanine (Vallon et al. 2005). Therefore, it might be speculated that apical KCNQ1, in addition to basolateral K^+^ channels, may also play a role in the repolarization of the luminal membrane in leaky epithelia, especially under relatively high‐substrate loads (Warth 2003; Warth and Barhanin 2003).

Against this background, a model may be offered in order to answer question 1: Due to changes in the composition of the jejunal digesta or due to changes in the hormonal status, the epithelial properties in lactating animals are changed substantially as indicated by the decrease in the basal *I*_sc_ and the decreased tissue conductance in lactating animals. The resulting higher transport activities for nutrients and ions, including a high activity of SGLT1, necessitate the induction of an apical K^+^‐conductance in order to maintain the apical to intracellular membrane potential. This K^+^‐secretion masks the increase in *I*_sc_ that was expected to occur when investigating jejunal tissue from lactating animals in Ussing‐chamber experiments.

The contradictions highlighted in question 2 may be discussed under 3a methodical aspect.

In tissues from dried‐off sheep, a significant increase in the *I*_sc_ after the mucosal addition of glucose was found, while no uptake was detectable in BBMV uptake experiments. The slight variations in the heights of the specific bands observed in the western blot experiments may offer an explanation for the putatively changed function at concurrently unchanged expression levels. The activity of SGLT1 can be modulated by both, activating and inactivating phosphorylation (Wright et al. 1997). It might be speculated that SGLT1 in dried off sheep is modified by such an inactivating phosphorylation which is removed immediately when glucose concentration is increased in the mucosal compartment of the Ussing‐chamber – a process that is impaired in BBMV lacking the cytosolic fraction. Future studies should address this point.

In summary, the results of this study confirm the thesis that changes in the intestinal glucose uptake are not solely explained by changes in the number of apical SGLT1 protein as a result of an increase in luminal substrate supply, but that additional factors such as posttranslational modifications resulting in changes in the turnover number could be involved. In addition, the voltage‐dependent action of intestinal nutrient transporters may play a role in the regulation of intestinal nutrient transport especially in ruminants, since the concept of feeding by pass starch significantly alters the composition of the jejunal digesta, resulting in marked changes of the epithelial properties that have to be considered during the conception and interpretation of further studies.

## Conclusions

Although sheep in early lactation compensate for the Ca loss mainly by increasing bone mobilization, gastrointestinal Ca absorption, especially across the ruminal epithelium, is stimulated to a certain degree. The observed differences in electrophysiological properties of ruminal and jejunal epithelia indicate an impact of either lactation and/or the respective feeding regime on ion transport. This might also explain the switch from electrogenic to electroneutral jejunal glucose transport observed in lactating sheep.

## Conflicts of Interest

The authors declare that there are no conflicts of interest.
